# Super-light Cu@Ni nanowires/graphene oxide composites for significantly enhanced microwave absorption performance

**DOI:** 10.1038/s41598-017-01529-2

**Published:** 2017-05-08

**Authors:** Xiaoxia Wang, Baoqin Zhang, Wei Zhang, Mingxun Yu, Liang Cui, Xueying Cao, Jingquan Liu

**Affiliations:** 1 College of Materials Science and Engineering; Institute for Graphene Applied Technology Innovation; Laboratory of Fiber Materials and Modern Textiles, the Growing Base for State Key Laboratory; Collaborative Innovation Center for Marine Biomass Fibers Materials and Textiles of Shandong Province, Qingdao University, Qingdao, 266071 China; 2 Shandong Institute of Nonmetal Materials, Jinan, 250031 China

## Abstract

Graphene oxide (GO) was rarely used as microwave absorption (MA) material due to its lower dielectric loss compared with reduced GO (RGO). However, the characteristics of low conductivity, light weight, and large surface area were beneficial to the impedance matching for absorbers already containing highly conductive metal materials. Cu@Ni nanowires are promising MA materials due to the desired dielectric loss from copper and excellent magnetic loss from nickel. However, the high density was an impediment to its further application. Combining Cu@Ni nanowires with GO should be an effective solution to decrease the absorber’s density and improve its MA properties. Herein, we demonstrated that Cu@Ni nanowires/GO composites exhibited enhanced MA capacities compared with Cu@Ni nanowires or GO alone, and the minimum reflection loss reached −42.8 dB at 16.9 GHz with a thickness of 2.1 mm. The enhanced MA performance mainly originated from good impedance matching, as a result of the addition of low conductivity of GO. To confirm this point, the MA performance of Cu@Ni nanowires/RGO was studied, and unsurprisingly, weak MA performance was obtained. Our work provides a new strategy to decrease the density, broaden the frequency band and tune MA performance of composites.

## Introduction

Recently, the increasing demand for microwave absorption (MA) materials with high efficiency, broad-bandwidth, and lightweight have attracted considerable attentions due to the serious electromagnetic interference (EMI) problems, which origin from the rapid advancement of electronic instruments, such as local area networks, electronic devices, and wireless communication tools^1, 2^. Although many new kinds of MA materials came forth constantly, achieving MA materials to meet the higher demands for excellent MA performance and low density still remains a forever challenge. Among all the MA materials, bimetallic core@shell or alloy nano-materials exhibited exciting MA performance owing to their multi-component structure, good impedance matching and strong interfacial polarization^3–5^ compared with one-component material.

Bimetallic Cu@Ni hybrid composites have been proven to be promising MA materials due to the desired dielectric loss from copper and excellent magnetic loss from nickel. For example, dendrite-like NiCu alloy^6^ obtained at 140 °C by hydrothermal process exhibited outstanding electromagnetic wave absorption properties, and the minimum reflection loss (RL) value was −31.13 dB at 14.3 GHz. CuNi nanowires and nanospheres^7^ were prepared by a chemical method and the minimum RL values were −22.9 dB at 12.6 GHz and −21.4 dB at 9.1 GHz, respectively. The simple operating procedure and mild reaction condition guaranteed the easy obtaining of Cu@Ni composites. In addition, compared with Cu or Ni alone, Ni shell and Cu core of Cu@Ni composites could effectively prevent the oxidation of Cu, thus could provide stable and higher dielectric loss. However, in practical application, the high density and magnetic aggregation for Cu@Ni composites are the critical challenges in MA field^8–10^. Therefore, how to reduce the density and further improve the MA property of Cu@Ni composites are still in urgent demand.

Carbon-based materials are promising candidates as lightweight component to decrease the density and increase the dielectric loss for MA composites. Among various carbon materials, low-dimensional carbon materials, like carbon nanotubes (CNTs)^11^ and reduced graphene oxide (RGO)^12^ were widely investigated as MA materials owing to their high dielectric loss, large surface areas, obvious anisotropy, good thermal and mechanical properties^13, 14^, while graphene oxide (GO) was rarely used due to its low conductivity^15^. It is well known low conductivity corresponds to low dielectric loss, and then leads to poor MA performance. However, for some metallic, bimetallic core@shell or alloy MA materials with high conductivity, like Cu@Ni composites, adding CNTs or RGO sheets can easily induce the impedance mismatching between too high permeability (ε_r_) and too low permittivity (μ_r_)^16^. As we know, poor impedance matching would induce a strong reflection of the electromagnetic wave^17^, and then further compromise the MA performance. Therefore, in this case, compared with high conductivity of Cu@Ni composites, the low conductivity of GO should become an excellent regulator to adjust the degree of complementarity between ε_r_ and μ_r_ in Cu@Ni/GO composites, and then enhance the MA performance.

It is well known that the low conductivity of GO comes from the hydroxyl, expoxy, carboxylic functional groups on its basal planes and edges. These functional groups can act as polarization centers to attenuate electromagnetic wave by electronic polarization, ionic polarization, dipole polarization^18^. In addition, the large surface area of GO will be beneficial to induce multiple scattering^19^, which supplies an effective way to transfer the electromagnetic energy into heat energy. From the points mentioned above, GO would be a promising candidate as lightweight filler for MA composites.

Herein, we demonstrate the facile fabrication of lightweight Cu@Ni nanowires/GO composites for MA performance. The reason for designing Cu@Ni nanowires (Cu@Ni NWs) as MA material is to utilize the synergistic effect between the Cu and Ni to achieve the enhanced MA performance. In addition, the large aspect ratio of nanowires is beneficial for the generation of polarization, which could significantly improve the MA performance^17, 20^. In this work, firstly, bimetallic Cu@Ni NWs have been successfully synthesized. In order to reduce the density and improve the MA performance of the absorber, GO was employed innovatively to prepare Cu@Ni NWs/GO composites, and the mass ratios of Cu@Ni NWs and GO were 1:0.5, 1:1, and 1:2, respectively (denoted as Cu@Ni NWs/GO (1:0.5), Cu@Ni NWs/GO (1:1), and Cu@Ni NWs/GO (1:2), respectively). Then the MA performance and mechanisms of the as-prepared Cu@Ni NWs/GO composites were investigated in detail, and the results revealed that the good complement between relative permittivity and permeability should be the main contribution to the enhanced MA performance of Cu@Ni NWs/GO composites.

## Results and Discussion

**Figure 1:**
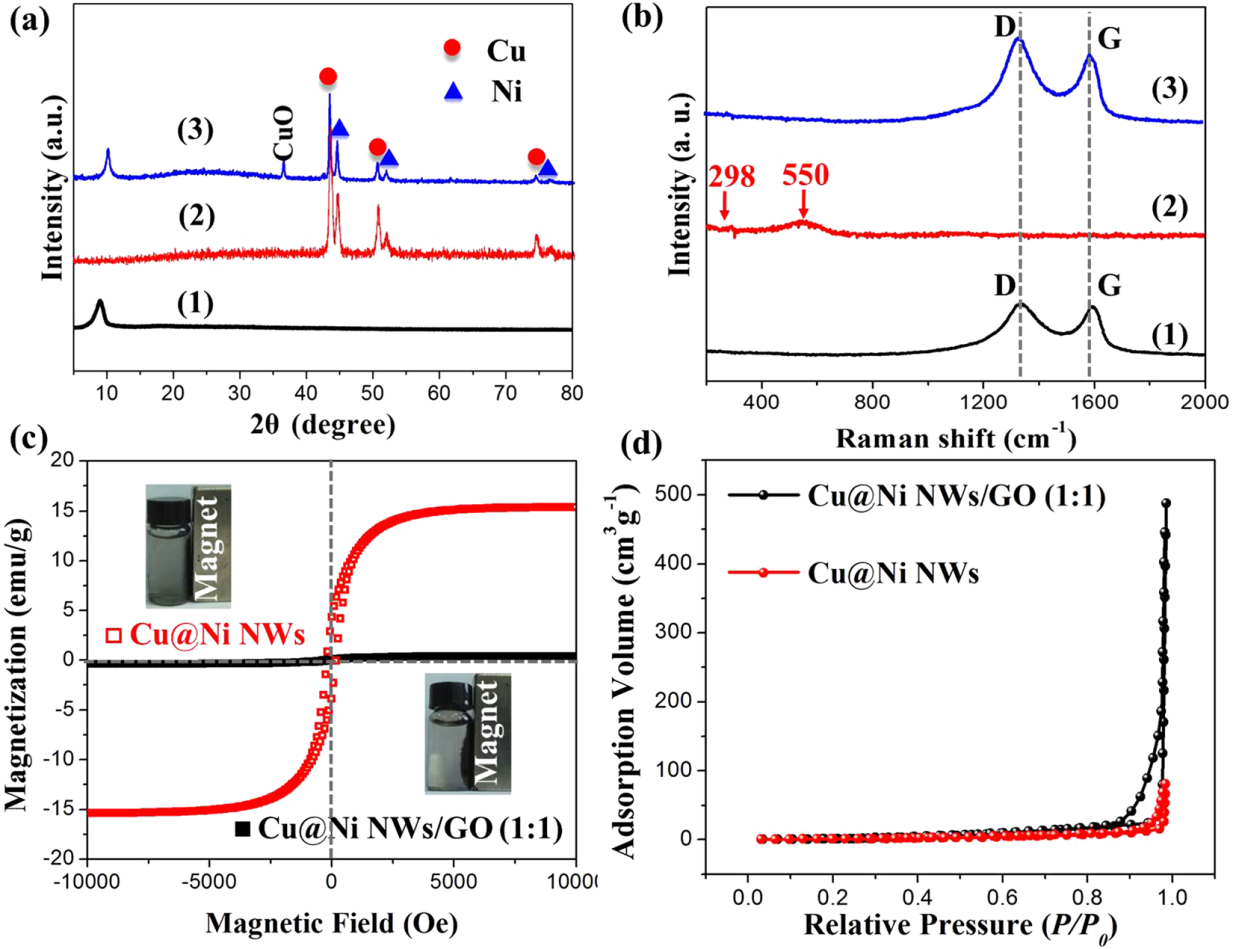
(**a**) XRD patterns of GO (1), Cu@Ni NWs (2), and Cu@Ni NWs/GO (1:1) (3). (**b**) Raman spectra of GO (1), Cu@Ni NWs (2), and Cu@Ni NWs/GO (1:1) (3). (**c**) Hysteresis loops of Cu@Ni NWs and Cu@Ni NWs/GO (1:1) measured at 300 K. The insets of upper left and the bottom right represent the photographs of Cu@Ni NWs and Cu@Ni NWs/GO (1:1) suspensions after application of magnetic field. (**d**) Nitrogen adsorption–desorption isotherm curves of the Cu@Ni NWs and Cu@Ni NWs/GO (1:1).

XRD analysis was used to determine the phase structure of GO, Cu@Ni NWs, and Cu@Ni NWs/GO (1:1). As shown in Fig. 1a, the X-ray pattern of GO shows a strong peak at about 10°, corresponding to (002) reflection peak of C, which was lower than the diffraction peak of graphite (2θ = 26°) due to the oxidation process. The characteristic peaks at 43.3°, 50.4°, 74.1° corresponded to (111), (200), (220) planes of face centered cubic (fcc) metallic Cu (JCPDS card no. 04-0836). The characteristic peaks of metallic Ni were also observed at 44.5°, 51.8°, and 76.4°, which could be indexed to (111), (200) and (220) crystalline planes of fcc Ni (JCPDS card no. 04-0850), respectively. The XRD results indicated that copper ions and nickel ions were reduced into metal Cu and Ni, respectively. It should be pointed out that CuO was observed in Cu@Ni NWs/GO (1:1), which indicated that oxidization was occurred due to the existence of oxygenous groups on GO.

Raman spectroscopy was used to study the carbon materials. Figure 1b shows the Raman spectra of GO, Cu@Ni NWs, and Cu@Ni NWs/GO (1:1). It could be seen that GO and Cu@Ni NWs/GO (1:1) exhibited characteristic D band at 1349 cm^−1^ and G band at 1587 cm^−1^, which could correspond to the disorder in the structure and the in-plane displacement of carbon atoms in hexagonal carbon sheets, respectively^14, 21^. The I_D_/I_G_ intensity ratio of Cu@Ni NWs/GO (1:1) was higher than GO, which demonstrated more defects were existed in the structure of Cu@Ni NWs/GO (1:1). The Raman spectrum of Cu@Ni NWs exhibited CuO (298 cm^−1^) and NiO (550 cm^−1^) peaks^22^.

The magnetic performances of Cu@Ni NWs/GO (1:1) and Cu@Ni NWs were investigated at room temperature (Fig. 1c). The saturation magnetization values of Cu@Ni NWs/GO (1:1) and Cu@Ni NWs were 0.40 and 15.35 emu g^−1^, respectively. This result demonstrated that the magnetic performance of Cu@Ni NWs/GO (1:1) decreased dramatically compared with Cu@Ni NWs alone due to the addition of the nonmagnetic GO.

The nitrogen adsorption and desorption measurements were employed to investigate the Brunauer-Emmett-Teller (BET) surface area of Cu@Ni NWs/GO (1:1) and Cu@Ni NWs. As shown in Fig. 1d, the BET surface area of Cu@Ni NWs/GO (1:1) is 19.65 m^2^/g, which is much higher than that of Cu@Ni NWs (6.08 m^2^/g). Therefore, GO provides a large surface area for Cu@Ni NWs/GO (1:1) composites, which can greatly improve the chance of penetration for electromagnetic wave into the absorber.

**Figure 2:**
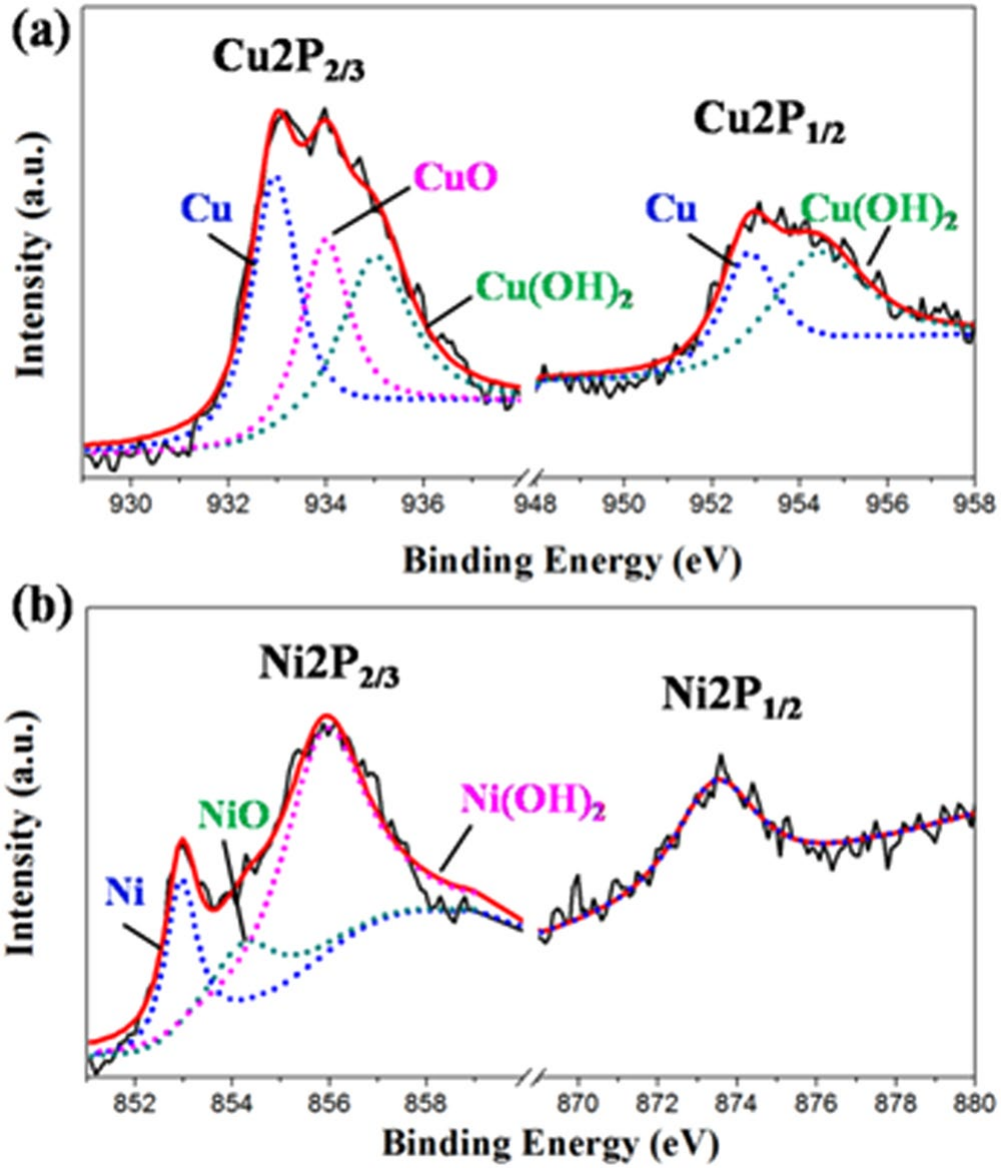
The XPS spectra of Cu@Ni NWs. Cu 2p (**a**) and Ni 2p (**b**).

The XPS data could provide sensitive information about the surface chemical composition of Cu@Ni NWs. In the XPS spectra (Fig. 2a), two Cu peaks at binding energies of 932.7 and 952.8 eV were observed, which corresponded to Cu2p_3/2_ and Cu2p_1/2_, respectively. The existence of CuO (933.9 and 952.8 eV) indicated that copper on the surface region could be oxidized easily at room temperature^23^. Cu(OH)_2_ (935 and 954.4 eV) was also appeared due to the presence of NaOH in the synthesis process. The peaks at 852.9 eV and 873.6 eV could be assigned to Ni2p_3/2_ and Ni2p_1/2_, respectively (Fig. 2b)^24^. The spectrum of Ni2p_3/2_ could be deconvoluted into three peaks, Ni (852.5 eV), NiO (854.3 eV), and Ni(OH)_2_ (856 eV), respectively, which displayed that Ni was partially oxidized on the surface region, too. Therefore, the XPS spectra demonstrated that Cu@Ni nanowires were partially oxidized at the surface layer due to the synthetic process and exposure to air.

**Figure 3:**
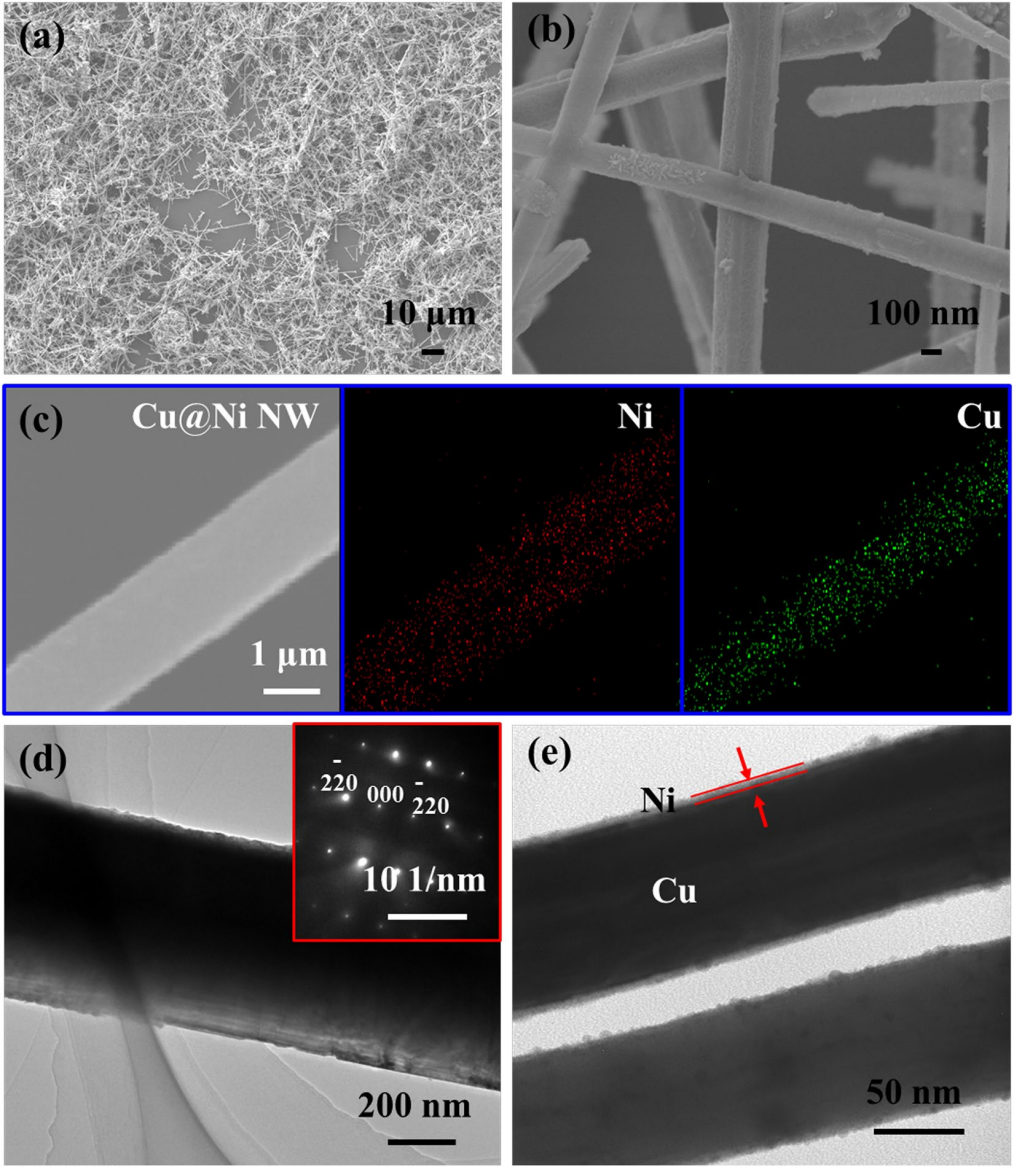
(**a**,**b**) SEM images of Cu@Ni NWs with different magnifications. (**c**) Cu@Ni NW and corresponding elemental mapping images of Ni and Cu. TEM images of Cu NW (**d**) and Cu@Ni NWs (**e**). The inset of Fig. 3(d) is the SAED pattern of Cu NWs.

In order to demonstrate that Cu@Ni NWs were formed with a structure of Cu-rich core and Ni-rich shell, SEM (Fig. 3a and b), EDS line-scan analysis and mapping (Fig. 3c), and TEM (Fig. 3d and e) were performed, respectively. As shown in Fig. 3a and b, the Cu@Ni NWs with high length-diameter ratios were synthesized successfully, and the corresponding elemental mapping images on an individual nanowire clearly indicated that Ni element existed at the wire edge, while Cu element mainly concentrated in the wire core (Fig. 3c). The TEM and corresponding SAED images (Fig. 3d) of Cu NW demonstrated that the crystal growth of Cu core was along the 〈110〉 direction^22^. The TEM image (Fig. 3e) of Cu@Ni NWs clearly exhibited a sheathed structure, and the thickness of Ni shell was about 8 nm.

**Figure 4:**
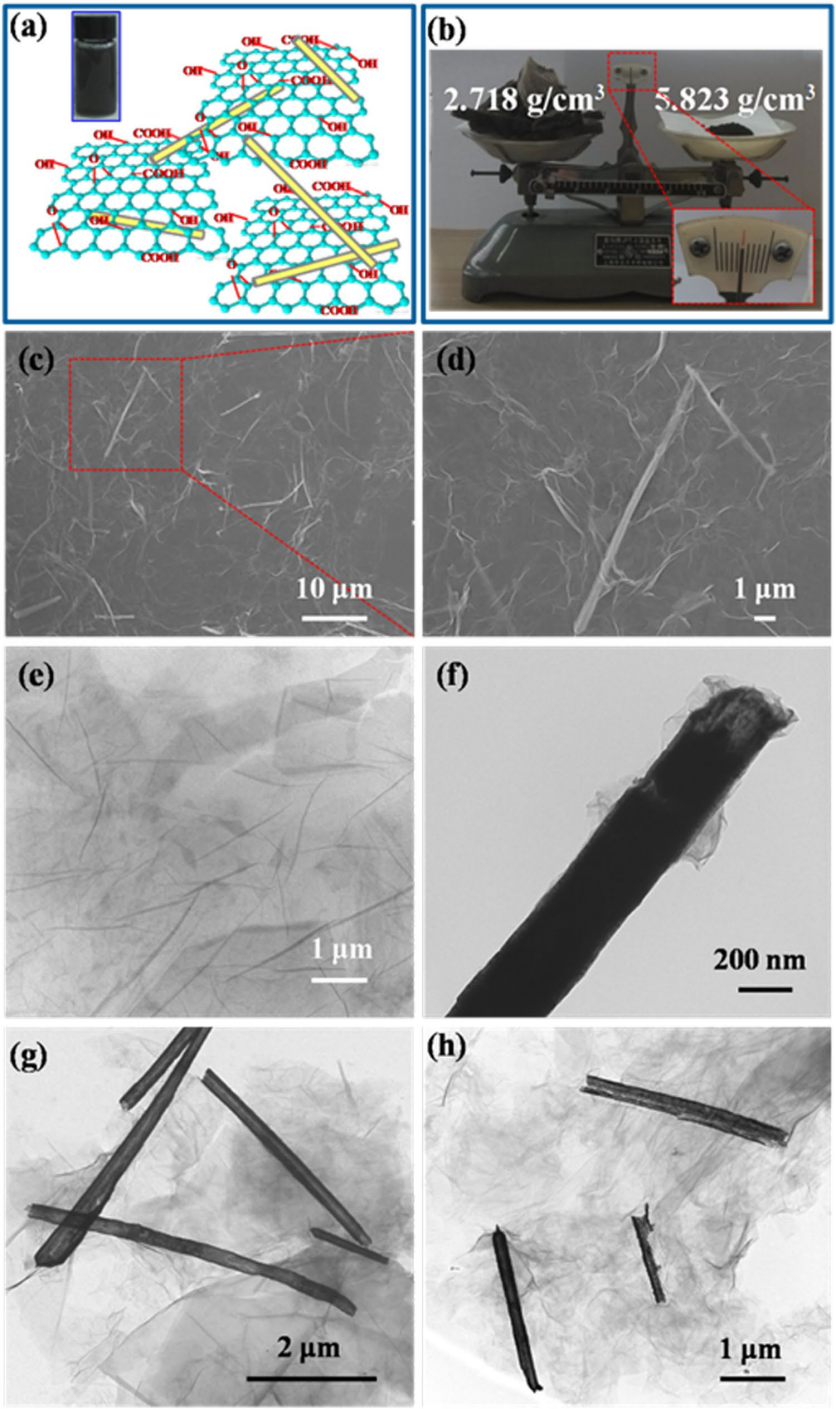
(**a**) Illustration of the Cu@Ni NWs/GO composites, and the photograph of Cu@Ni NWs/GO (1:1) suspension (upper left). (**b**) The lyophilized Cu@Ni NWs/GO (1:1) (left) and Cu@Ni NWs (right) with the same mass on the balance. (**c**,**d**) SEM images of Cu@Ni NWs/GO (1:1) with different magnifications. TEM images of GO (**e**), Cu@Ni NWs/GO (1:0.5) (**f**), Cu@Ni NWs/GO (1:1) (**g**), and Cu@Ni NWs/GO (1:2) (**h**).

GO sheets can disperse well in the aqueous medium and form composites with Cu@Ni NWs via interaction with the considerable amounts of hydroxyl, epoxide, carbonyl functional groups on GO (Fig. 4a). The as-prepared Cu@Ni NWs/GO (1:1) (density: 2.718 g/cm^3^) (Fig. 4b) was lightweight and exhibited a more fluffy structure compared with Cu@Ni NWs (density: 5.823 g/cm^3^). The homogeneous distribution of Cu@Ni NWs in the Cu@Ni NWs/GO (1:1) composites was evidenced by SEM image (Fig. 4c), and the corresponding amplified image (Fig. 4d) indicated that the Cu@Ni NWs were uniformly wrapped by the GO sheets. TEM was used to determine the morphology and the distribution of Cu@Ni NWs in the composites. As shown in Fig. 4e, the GO sheets were large, flat and almost transparent under electron beam. After the addition of GO, the Cu@Ni NWs were all coated by GO sheets (Fig. 4f,g and h). The higher the mass ratio of GO to Cu@Ni NWs, the more GO sheets were wrapped on the Cu@Ni NWs.

The microwave absorption (MA) properties of the Cu@Ni NWs/GO composites could be evaluated by the reflection loss (RL) value. Based on the transmission line theory, the value of RL of electromagnetic wave (perpendicular incidence), which depends on the parameters of relative permittivity, permeability, and the thickness of the absorber, can be derived from the following equations (1) and (2)^25, 26^.

1 $$RL({\rm{dB}})=20\,{\rm{log}}| \frac{{Z}_{in}\,-\,{Z}_{0}}{{Z}_{in}+{Z}_{0}}| $$

Where Z_0_ and Z_in_ are the impedance of free space and the input characteristic impedance, respectively, and Z_in_ can be expressed as below^27, 28^:

2 $${Z}_{in}={Z}_{0}\sqrt{\frac{{\mu }_{r}}{{\varepsilon }_{r}}}\,tan\,{\rm{h}}({\rm{j}}\frac{2\pi fd}{c}\sqrt{{\mu }_{r}{\varepsilon }_{r}})$$

where d, c, ƒ are the absorber’s thickness, the velocity of light, and the frequency of the electromagnetic waves, respectively. The real (ɛ′, μ′) and imaginary (ɛ′′, μ′′) parts of the complex relative permittivity and permeability can be measured by an Agilent N5244A vector network analyzer. Using the following equations (3) and (4)^29^, the values of ɛ_r_ and μ_r_ were obtained.

3 $${{\rm{\varepsilon }}}_{{\rm{r}}}={{\rm{\varepsilon }}}^{{\prime} }\,-\,{\rm{j}}{{\rm{\varepsilon }}}^{{\prime\prime} }$$

4 $${{\rm{\mu }}}_{{\rm{r}}}={{\rm{\mu }}}^{{\prime} }\,-\,{\rm{j}}{{\rm{\mu }}}^{{\prime\prime} }$$

Usually when RL value is less than −10 dB, more than 90% of all the incident electromagnetic waves are absorbed. This is a threshold value required for materials as suitable absorbers^30^.

**Figure 5:**
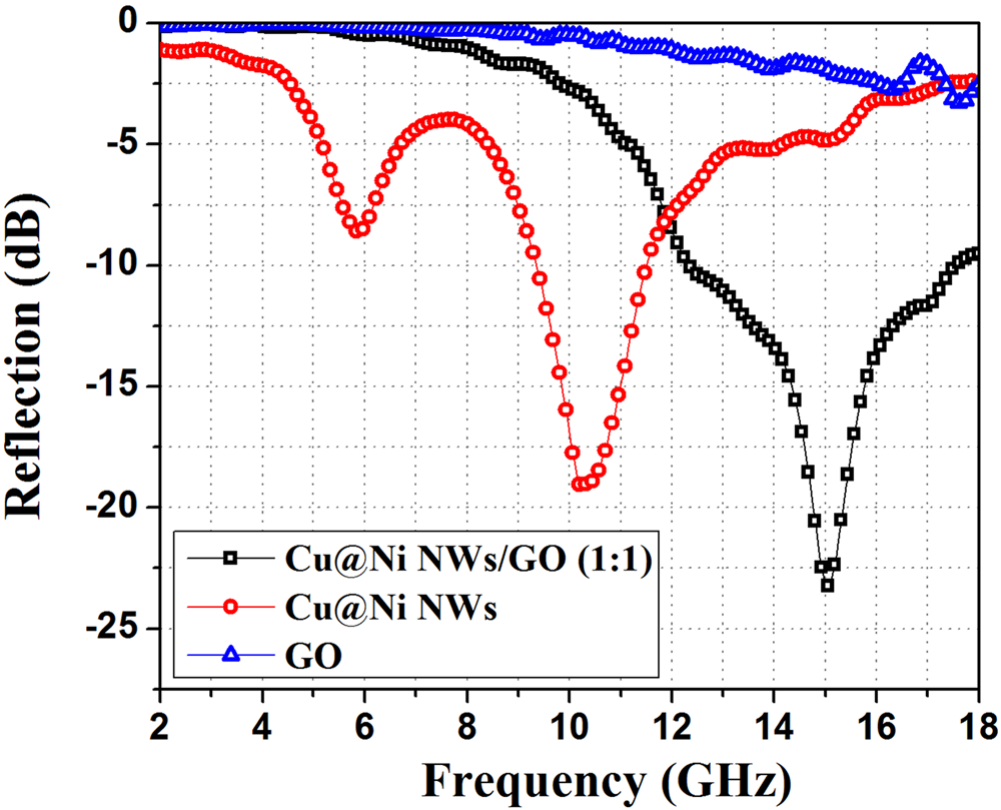
The RL curves of the Cu@Ni NWs/GO (1:1), Cu@Ni NWs, and GO samples in paraffin matrix with thickness of 2.5 mm in the frequency range of 2.0–18.0 GHz.

MA performances of Cu@Ni NWs/GO (1:1), Cu@Ni NWs, and GO were first investigated with a thickness of 2.5 mm in the frequency range of 2.0–18.0 GHz. As shown in Fig. 5, Cu@Ni NWs/GO (1:1) presented significantly enhanced MA capacity compared to Cu@Ni NWs or GO alone. The minimum RL value of Cu@Ni NWs/GO (1:1) was −23.08 dB at 15.0 GHz and the RL values were less than −10 dB in the frequency range of 12.4–17.6 GHz, corresponding to a bandwidth of 5.2 GHz. However, for Cu@Ni NWs, the minimum RL value was −19.07 dB at 10.3 GHz and the RL values were less than −10 dB in the range of 9.4–11.5 GHz, corresponding to a bandwidth of only 2.1 GHz. This demonstrated that the MA performance was improved and the bandwidth was obviously broadened for Cu@Ni NWs/GO (1:1) due to the addition of GO.

**Figure 6:**
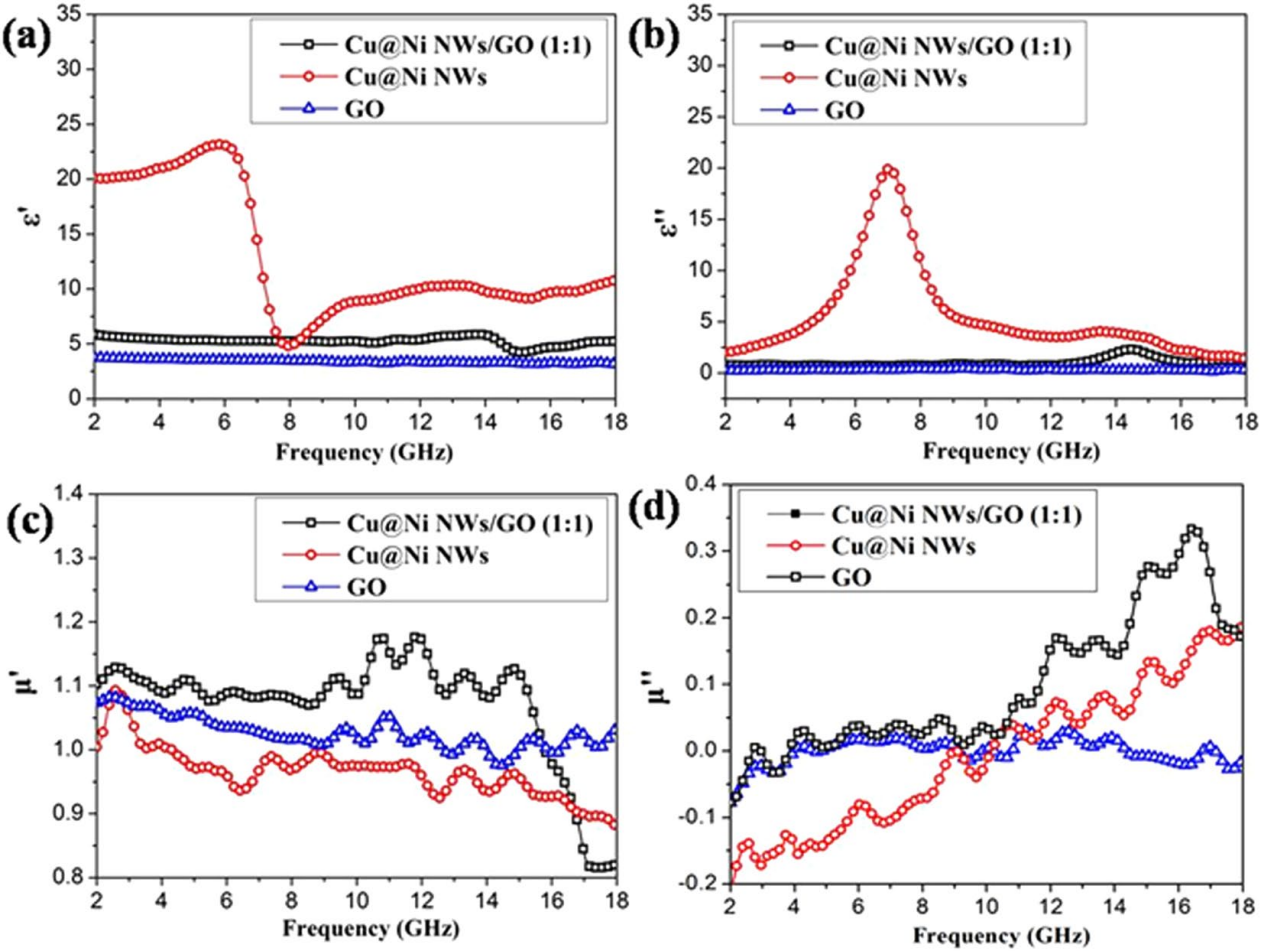
(**a**,**b**) Real (ε′) and imaginary (ε′′) parts of permittivity and (**c**,**d**) real (μ′) and imaginary (μ′′) parts of permeability for Cu@Ni NWs/GO (1:1), Cu@Ni NWs, and GO in the frequency range of 2.0–18.0 GHz.

In order to investigate the possible reasons why the Cu@Ni NWs/GO (1:1) composites exhibited enhanced MA performance than Cu@Ni NWs or GO alone, the real and imaginary parts of relative permittivity (ε′ and ε′′) and permeability (μ′ and μ′′) of samples were investigated in the 2.0–18.0 GHz frequency (Fig. 6). Obviously, Cu@Ni NWs showed much higher ε′ and ε′′ values (Fig. 6a and b) than Cu@Ni NWs/GO (1:1) and GO in the most range of frequency due to the high conductivity of Cu core. The resonance peak at about 7 GHz was observed for Cu@Ni NWs, which should be associated with the interfaces between Cu and Ni^31^. After mixing with GO, severely decreases in both ε′ and ε′′ values were observed for Cu@Ni NWs/GO (1:1), which should be attributed to the surface coating of GO on the Cu@Ni NWs. From the free electron theory ε′′ could be described as equation (5)^32, 33^:

5 $${{\rm{\varepsilon }}}^{{\prime\prime} }\approx 1/2{{\rm{\varepsilon }}}_{0}{\rm{\pi }}{\rm{\rho }}f$$

where ε_0_ is the permittivity of a vacuum, ρ is the resistivity, and ƒ is the frequency of the microwave. It can be concluded that the high electronic conductivity can lead to the high ε′′ value. Therefore, the conductivity of Cu@Ni NWs is the highest, followed by the Cu@Ni NWs/GO (1:1) and GO. However, too high ε′′ means that the relative permittivity and permeability are out of balance, which might be induced the fact that the RL values of Cu@Ni NWs/GO (1:1) were higher than Cu@Ni NWs.

Figure 6c and d show the complex variation of μ′ and μ′′ values of the samples. The low μ′ and μ′′ values of Cu@Ni NWs/GO (1:1) and Cu@Ni NWs should be originated from the less content of nickel. The Cu@Ni NWs/GO (1:1) showed the higher μ′ values than Cu@Ni NWs and GO in the frequency of 2.0–15.7 GHz, and the μ′′ values of Cu@Ni NWs/GO (1:1) were also higher than Cu@Ni NWs and GO, especially in the range of 9.4–18.0 GHz. This might be another reason why Cu@Ni NWs/GO (1:1) showed higher RL values than Cu@Ni NWs. The μ′ and μ′′ values of Cu@Ni NWs showed more fluctuation, which might be due to natural resonance^34^. It is noteworthy that many μ′′ values of the imaginary permeability of samples were negative, which indicated that the magnetic energy had been radiated out from the samples and transferred into the electric energy^35^.

**Figure 7:**
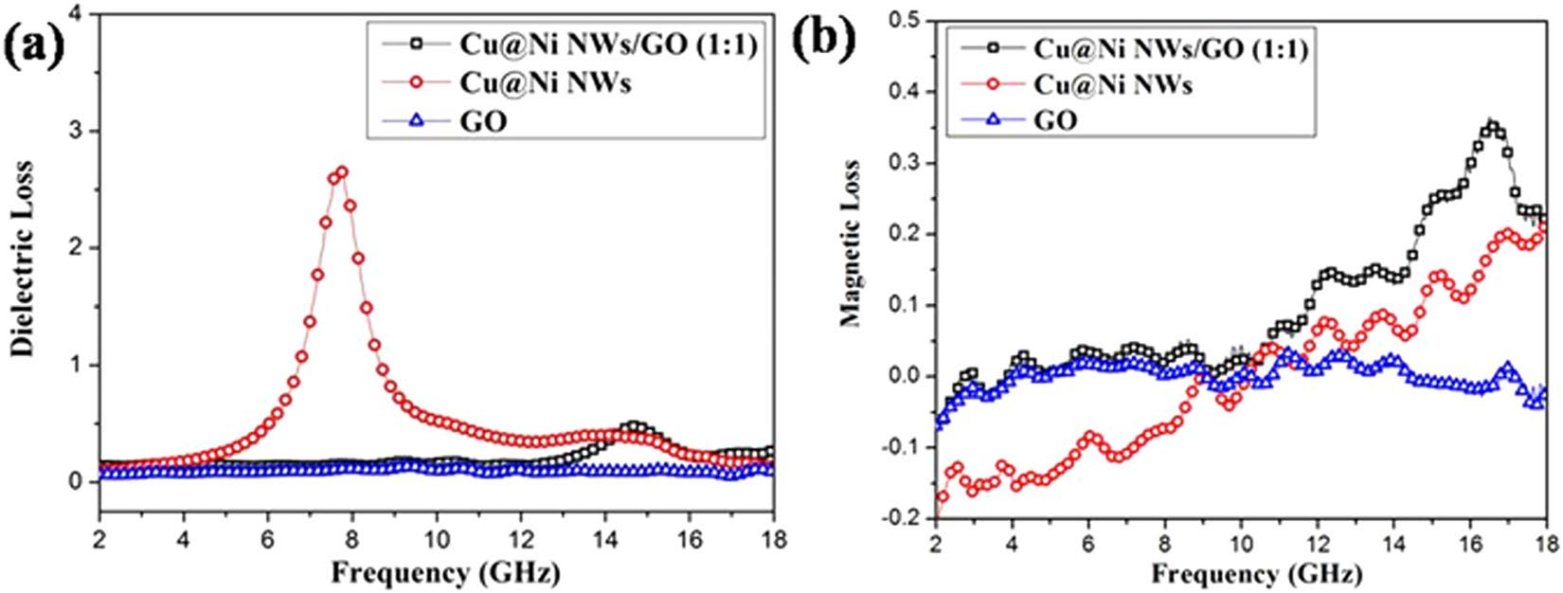
Dielectric loss (**a**) and magnetic loss (**b**) of Cu@Ni NWs/GO (1:1) composite, Cu@Ni NWs, and GO.

Dielectric loss (tanδ_e_ = ɛ′′/ɛ′) and magnetic loss (tanδ_μ_ = μ′′/μ′) are two major contributions to MA capacity^36, 37^. The tanδ_e_ and tanδ_μ_ of Cu@Ni NWs/GO (1:1), Cu@Ni NWs and GO were investigated (Fig. 7). Most of the tanδ_e_ values of Cu@Ni NWs exceeded those of Cu@Ni NWs/GO (1:1) and GO (Fig. 7a), that is, the dielectric loss was the main way to attenuate the electromagnetic wave for Cu@Ni NWs. For tanδ_μ_ (Fig. 7b), Cu@Ni NWs/GO (1:1) exhibited higher tanδ_μ_ than Cu@Ni NWs and GO, especially in the range of 9.4–18.0 GHz. Therefore, a maximum value for the dielectric loss or the magnetic loss alone doesn’t guarantee the high MA capacity, which should be achieved based on the prerequisite of good impedance matching between the dielectric loss and magnetic loss.

**Figure 8:**
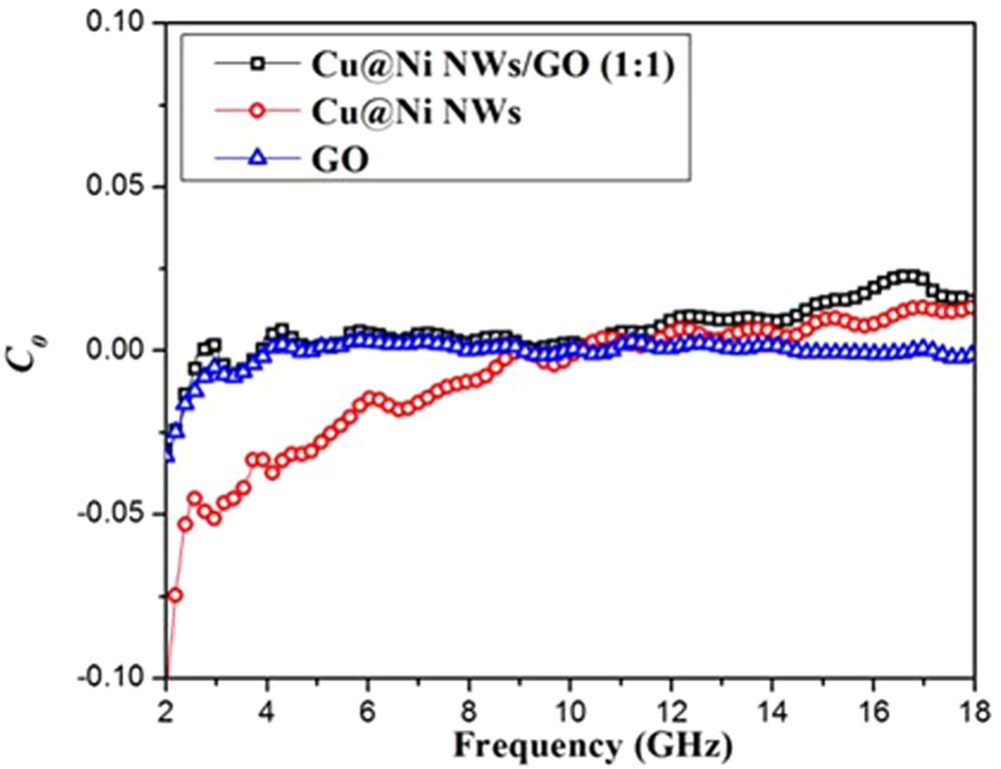
The values of *C*_*0*_ = μ′′(μ′)^−2^ƒ^−1^ versus the frequency for Cu@Ni NWs/GO (1:1), Cu@Ni NWs and GO in the frequency range of 2.0–18.0 GHz.

Eddy current loss is considered an important contributor to magnetic loss, which can be evaluated by equation (6)^38, 39^:

6 $${C}_{0}={{\rm{\mu }}}^{{\prime\prime} }{({{\rm{\mu }}}^{{\prime} })}^{-2}{f}^{-1}=2{{\rm{\pi }}{\rm{\mu }}}_{0}{{\rm{\sigma }}d}^{2}/3$$

where d, σ, μ_0_ are the diameter of the nanoparticle, the electric conductivity, and the permeability of vacuum, respectively. From this equation, if the magnetic loss mainly originates from the eddy current loss then the values of *C*_*0*_ will be constant when the frequency is changed. Figure 8 shows the relationship between the *C*_*0*_ values and the frequency. When ƒ was above 4.0 GHz, the values of *C*_*0*_ remained almost constant, which confirmed that the eddy current loss was the main contribution to magnetic loss for Cu@Ni NWs/GO (1:1), Cu@Ni NWs, and GO.

**Figure 9:**
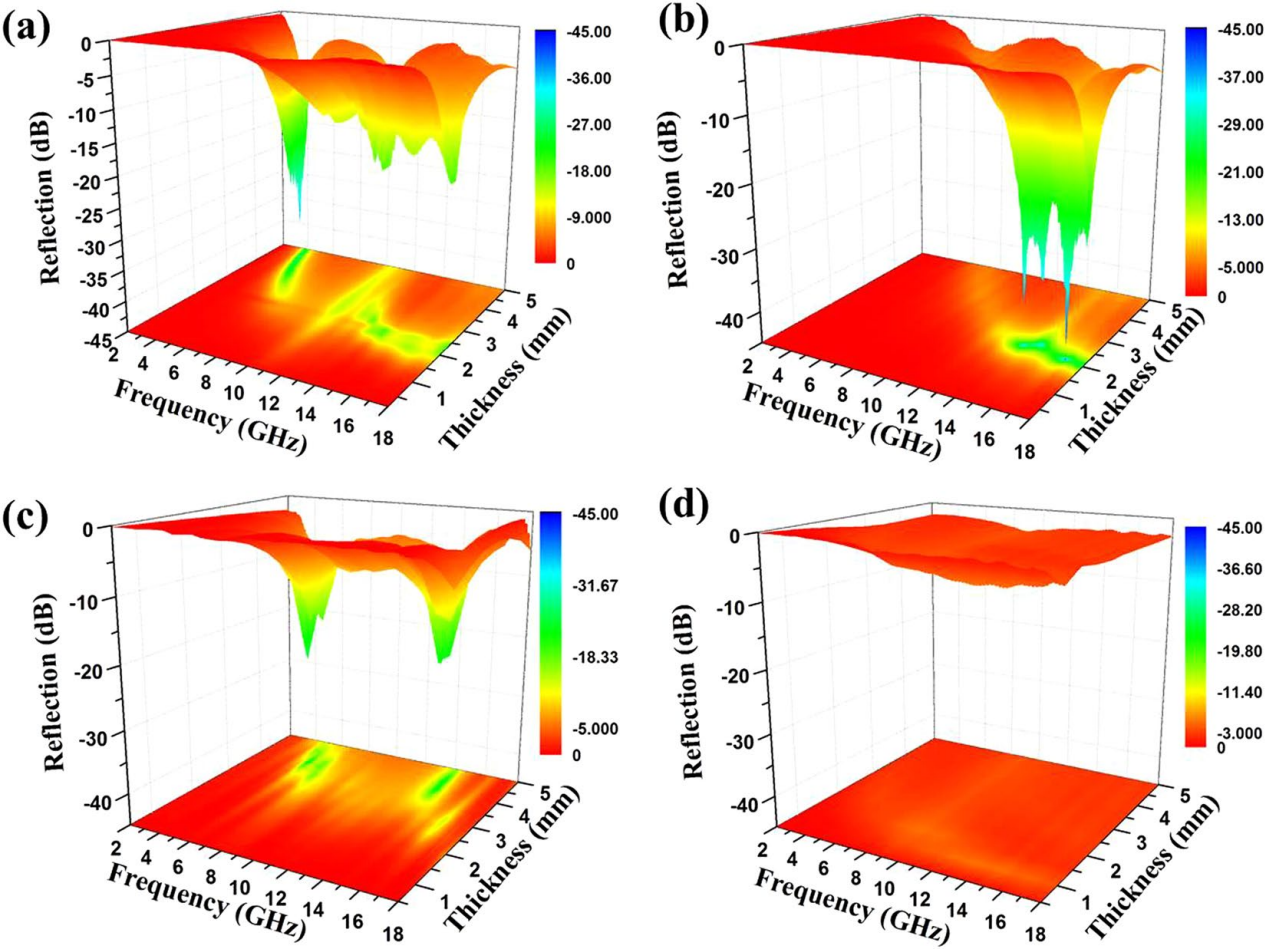
Three-dimensional representation of RL curves for Cu@Ni NWs/GO (1:0.5) (**a**), Cu@Ni NWs/GO (1:1) (**b**), Cu@Ni NWs/GO (1:2) (**c**), and Cu@Ni NWs/RGO (1:1) (**d**).

In order to further investigate the GO’s contribution to MA capacity and the thickness’s influence to MA characteristics of Cu@Ni NWs/GO, three-dimensional representation RL curves of Cu@Ni NWs/GO composites with different mass ratios of Cu@Ni NWs to GO (1:0.5, 1:1, and 1:2) under various thicknesses were achieved and the results were shown in Fig. 9a,b and c, respectively. It could be seen that, among the three samples, the mimimum RL value of −42.8 dB at 16.9 GHz was observed for Cu@Ni NWs/GO (1:1) with a thickness of 2.1 mm.

The minimum RL values for Cu@Ni NWs/GO (1:0.5) and Cu@Ni NWs/GO (1:2) were −38.2 and −26.07 dB at 4.2 and 5.6 GHz with the thickness of 4.6 and 4.0 mm, respectively. That is, too much Cu@Ni NWs or GO could not result in the best MA performance. In order to fully express the relationship among the RL values, the frequency and the thickness, two-dimensional curves derived from the three-dimensional curves (Fig. 9) of Cu@Ni NWs/GO (1:0.5), Cu@Ni NWs/GO (1:1), and Cu@Ni NWs/GO (1:2) under different thickness selected from 0.5 to 5 mm in the frequency range of 2.0–18.0 GHz are shown in Fig. S1a,b and c. For Cu@Ni NWs/GO composites, especially for Cu@Ni NWs/GO (1:1), the synergetic effect between Cu@Ni NWs and GO provided a MA performance boost, which mainly originated from the effective impedance matching.

To prove the point that the impedance matching played a very important role in the improvement of MA capacity for Cu@Ni NWs/GO composites, Cu@Ni NWs/reduced GO (Cu@Ni NWs/RGO) composites were prepared. The RGO reduced from GO by hydrazine^40^, and the mass ratio of Cu@Ni NWs to RGO was designed at 1:1 (denoted as Cu@Ni NWs/RGO (1:1)). Three-dimensional (Fig. 9d) and two-dimensional (Fig. S1d) representation RL curves of Cu@Ni NWs/RGO (1:1) composites under various thicknesses are exhibited. Obviously, large performance gap was observed for Cu@Ni NWs/GO (1:1) and Cu@Ni NWs/RGO (1:1) (the minimum reflection value of Cu@Ni NWs/RGO (1:1) <−5 dB). That is, when the mass ratios of Cu@Ni NWs to GO or RGO were the same, Cu@Ni NWs/GO exhibited much higher MA performance than Cu@Ni NWs/RGO. The MA capacity of Cu@Ni NWs/RGO was even worse than Cu@Ni NWs due to the excessively high conductivity generated by the combined action of RGO and Cu@Ni NWs. The impedance mismatching occurred between the too high permittivity and too low permeability, and then poor MA performance appeared. This result reinforced the point that though Cu@Ni NWs were the main contributions to the MA performance of composites, the contribution of GO could not be ignored. In addition, the complement between relative permittivity and permeability could be tuned upon the addition of GO.

**Figure 10:**
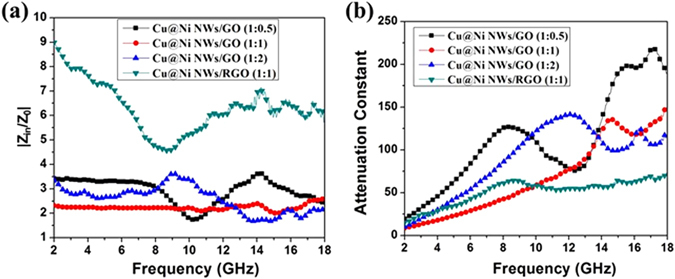
Impedance matching (**a**) and attenuation constant α (**b**) of Cu@Ni NWs/GO (1:0.5), Cu@Ni NWs/GO (1:1), Cu@Ni NWs/GO (1:2), and Cu@Ni NWs/RGO (1:1).

The characteristic of impedance matching could be evaluated by the value of |Z_in_/Z_0_| based on equation (2)^41, 42^. The closer that the value between composites (Z_in_) and free space (Z_0_) (|Z_in_/Z_0_|) is to 1, the better MA performance will be acquired. The values of |Z_in_/Z_0_| for Cu@Ni NWs/GO (1:0.5), Cu@Ni NWs/GO (1:1), Cu@Ni NWs/GO (1:2), and Cu@Ni NWs/RGO (1:1) were showed in Fig. 10a, which were calculated based on the parameters shown in Fig. S2. It was observed that the |Z_in_/Z_0_| values for Cu@Ni NWs/RGO (1:1) were obviously larger than 1, and higher than that for Cu@Ni NWs/GO (1:0.5), Cu@Ni NWs/GO (1:1), and Cu@Ni NWs/GO (1:2) in the frequency range of 2.0–18.0 GHz, which demonstrated that the impedance mismatching was occurred for Cu@Ni NWs/RGO (1:1). In addition, when ɛ_r_ = μ_r_, then Z_in_ = Z_0_, and the best impedance matching could be obtained. In Fig. S2, Cu@Ni NWs/RGO shows an obvious higher ɛ_r_ values than that of Cu@Ni NWs/GO. The huge gap between the too high permittivity and the too low permeability could cause the impedance mismatching for Cu@Ni NWs/RGO.

The integral attenuation property of penetrated electromagnetic waves could be evaluated by the attenuation constant (α), which could be expressed as equation (7) as below^39, 43^.

7 $$\alpha =\frac{\sqrt{2}\pi f}{c}\sqrt{({\mu }^{{\prime\prime} }{\varepsilon }^{{\prime\prime} }-{\mu }^{{\prime} }{\varepsilon }^{{\prime} })+\sqrt{{({\mu }^{\prime\prime}{\varepsilon }^{\prime\prime}-{\mu }^{{\prime} }{\varepsilon }^{{\prime} })}^{2}+{({\mu }^{{\prime} }{\varepsilon }^{\prime\prime}-{\mu }^{\prime\prime}{\varepsilon }^{{\prime} })}^{2}}}$$

The values of α for Cu@Ni NWs/GO (1:0.5), Cu@Ni NWs/GO (1:1), Cu@Ni NWs/GO (1:2), and Cu@Ni NWs/RGO (1:1) are shown in Fig. 10b. Obviously, Cu@Ni NWs/RGO (1:1) exhibited the lowest attenuation constant α in the frequency of 10.0–18.0 GHz. That is, compared with Cu@Ni NWs/GO, Cu@Ni NWs/RGO (1:1) showed worse integral attenuation property in the frequency of 10.0–18.0 GHz.

The MA performances of other mass ratio of Cu@Ni NWs to RGO (1:0.5 and 1:2) were investigated, too (denoted as Cu@Ni NWs/RGO (1:0.5) and Cu@Ni NWs/RGO (1:2)). The RL results (Fig. S3) demonstrated that all the Cu@Ni NWs/RGO samples exhibited poor MA performance. The dielectric loss and the magnetic loss of Cu@Ni NWs/RGO (1:0.5), Cu@Ni NWs/RGO (1:1), and Cu@Ni NWs/RGO (1:2) are shown in Fig. S4, which demonstrates that an obvious gap is existed between the high dielectric loss and the low magnetic loss^44^.

**Figure 11:**
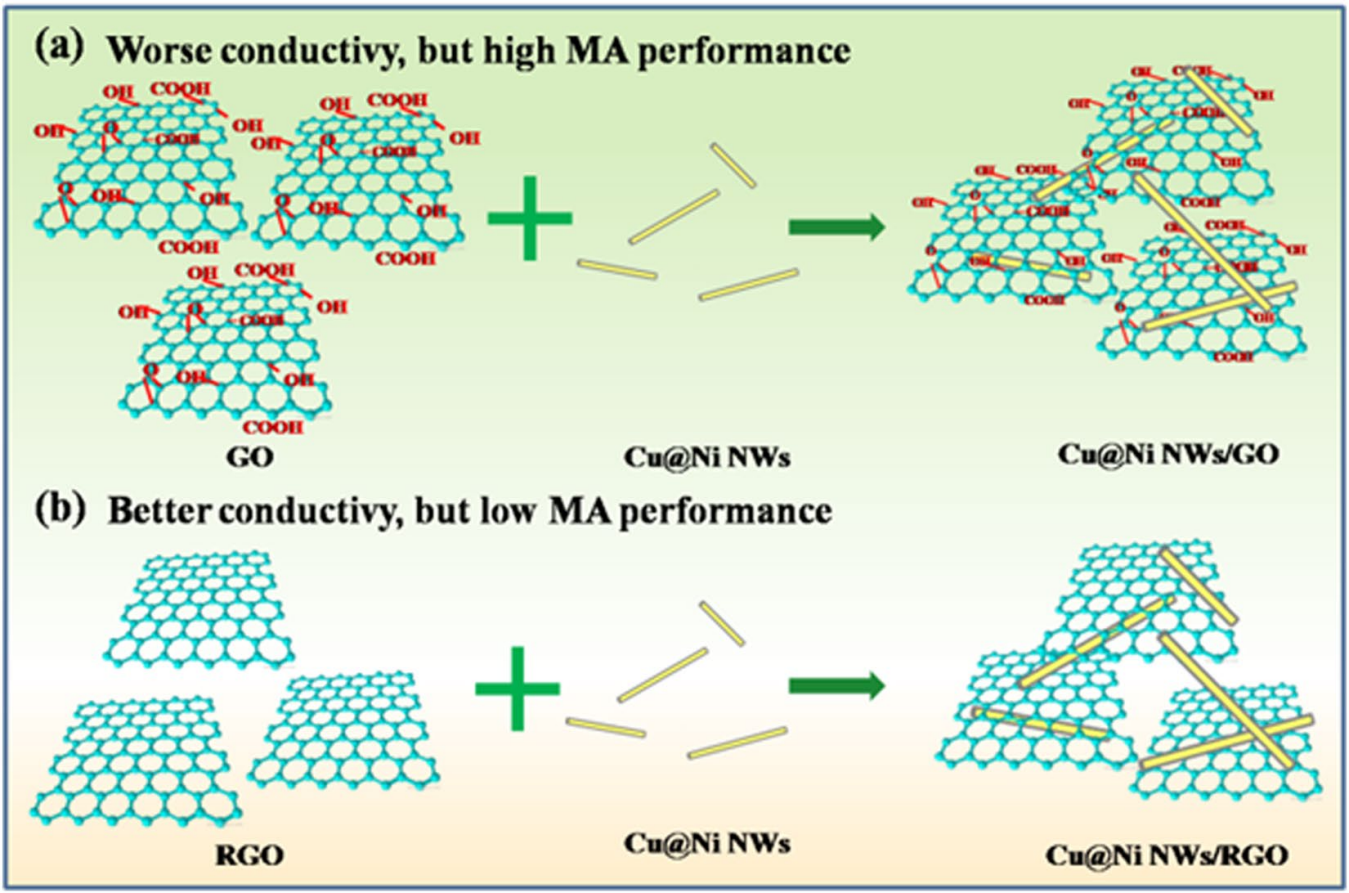
Schematic illustrations of the preparation process of Cu@Ni NWs/GO (**a**) and Cu@Ni NWs/RGO (**b**) composites.

Figure 11 illustrates the possible reasons why the Cu@Ni NWs/RGO composites showed an enhanced conductivity. It is obvious that for GO sheets, electrons traveling will be hindered by a lot of defects and groups, leading to the low conductivity. When GO is coated on the random networks of Cu@Ni NWs, the as-formed Cu@Ni NWs/GO composites exhibit low electrical property due to the increased junction resistances. However, when RGO sheets combine with Cu@Ni NWs, nanowires can bridge the RGO sheets to provide a new transport route for electron transfer and therefore improve the conductivity^45^. However, for Cu@Ni NWs/RGO composites the high conductivity will induce the impedance mismatching. According to equation (5), we know that the high electronic conductivity can lead to the high ε′′ value. So the relationship between the values of the maximum |RL| and the corresponding ε′′ values can reflect the relationship between the MA performance and the conductivity. In current paper, Cu@Ni NWs/RGO exhibited the higher ε′′ values and lower |RL| values compared with Cu@Ni NWs/GO because of the higher electronic conductivity of Cu@Ni NWs/RGO than Cu@Ni NWs/GO (Fig. S5). That is, simply improving the dielectric loss is not necessarily guarantee the excellent MA performance for Cu@Ni NWs/RGO composites^44^.

In addition, interfacial polarization is another way to convert electromagnetic energy into heat energy, and it usually exists in heterogeneous system due to the different dielectric constants^46^. In Cu@Ni NWs/GO composites, the multi-interfaces and double junctions (Cu@Ni, Ni@GO) could account for the loss mechanisms. Multiple scattering produced by large surface area of GO was another favorable factor to increase the loss of electromagnetic energy^19^. Therefore, the multi-interfaces, double junctions, and multiple scattering are all contributors to the enhanced MA capacity of Cu@Ni NWs/GO composites.

## Conclusions

Cu@Ni NWs have been successfully synthesized via a simple “one step reduction” method, and then utilized for the preparation of Cu@Ni NWs/GO composites. The MA performance of Cu@Ni NWs/GO composites were investigated based on the transmission/reflection coaxial line method. Compared with Cu@Ni NWs/RGO, Cu@Ni NWs or GO alone, Cu@Ni NWs/GO exhibited the significantly enhanced MA performance, which mainly came from the efficient impedance matching, owning to the addition of GO. Furthermore, both the interfacial polarization and multiple scattering were favorable factors to the improvement of MA capacity. Therefore, the Cu@Ni NWs/GO composites could be used as promising lightweight and efficient microwave absorbers.

## Methods

### Chemicals

Nickel nitrate [Ni(NO_3_)_2_ **·** 6H_2_O], copper nitrate [Cu(NO_3_)_2_ **·** 3H_2_O] were purchased from Sinopharm Chemical Reagent Co., Ltd. Sodium hydroxide, urea, ethanediamine (EDA, 99 wt%), and hydrazine (80 wt%) were purchased from Aladdin Reagent Co., Ltd., and deionized water was employed for the preparation of all solutions. GO was synthesized by the modified Hummers method4^47^, and then dispersed in deionized water by ultrasonication bath for 4 h at 25 °C to afford a uniform suspension with a concentration of 0.5 mg/mL.

### Synthesis of Cu@Ni NWs

The Cu@Ni NWs were synthesized by “one-pot” synthesis method^48^ as described below: 30 mL NaOH (7 M) solution was added in a plastic beaker, then 0.13 mL copper nitrate solution (0.5 M) and 0.07 mL nickel nitrate solution (0.5 M) were added in the beaker, the resulting mixture was stirred for about 5 min. After that, EDA (0.15 mL) and hydrazine (0.025 mL) were joined and stirred for another 5 min, then heated in water bath for 1 h at 80 °C. The sediment was centrifuged and washed with deionized water for several times, and then dried by lyophilized method.

### Synthesis of Cu@Ni NWs/GO composites

The synthesis of Cu@Ni NWs/GO composites were achieved by simple blending of Cu@Ni NWs with GO through ultrasonic method. In order to determine the effect of GO, different mass ratios of Cu@Ni NWs to GO were designed at 1:0.5, 1:1, and 1:2, respectively. Taking the mass ratio of 1:1 as an example, the procedures of Cu@Ni NWs/GO composites were described below: 20 mg of dried Cu@Ni powder was added to 40 mL GO suspension (0.5 mg/mL) and sonicated for 4 h at 25 °C to form a uniform suspension, and then the products were centrifugated and washed with deionized water for several times. Finally the Cu@Ni NWs/GO composites were obtained by lyophilized method. The Cu@Ni NWs/RGO composites were obtained by the same method as Cu@Ni NWs/GO, except using RGO instead of GO.

### Structural characterizations and microwave absorption measurements

XRD patterns were taken on a Rigaku D—MAX 2500/PC using Cu Kα radiation (λ = 1.54056 Ǻ). The morphologies and structures of Cu@Ni NWs and Cu@Ni NWs/GO composites were collected by a field emission scanning electron microscopy (FESEM, JEOL JSM-6700F) and transmission electron microscope (TEM, JEOL JEM-2100). Raman Spectra of samples were measured using a LabRAM HR confocal Raman system with 532 nm diode laser excitation at room temperature. Magnetic hysteresis loops of room temperature were measured on a SQUID susceptometer (Quantum Design Co.) The surface area was calculated using the Brunauer–Emmett–Teller (BET) method based on the adsorption data. The chemical composition of the surface region of Cu@Ni NWs was conducted using X-ray photoelectron spectroscopy (XPS) with an Al Kα radiation source of Thermo ESCALAB 250XI. The densities of Cu@Ni NWs and Cu@Ni NWs/GO (1:1) were measured by density type analyzer (TD-1200).

For the electromagnetic parameter measurements, the samples were firstly prepared with 30 wt% of as-obtained composites and 70 wt% paraffin ceresin, and then the resulting mixtures were pressed into a cylindrical shaped compact with an inner and outer diameter of 3.04 mm and 7.00 mm, respectively. The complex relative permittivity and permeability of the composites were obtained based the transmission/reflection coaxial line method from 2.0 to 18.0 GHz using an Agilent N5244A vector network analyzer.

## Electronic supplementary material

Super-light Cu@Ni nanowires/graphene oxide composites for significantly enhanced microwave absorption performance

## References

[1] Zhao S (2016). Alternate nonmagnetic and magnetic multilayer nanofilms deposited on carbon nanocoils by atomic layer deposition to tune microwave absorption property. Carbon.

[2] Wang G (2014). High densities of magnetic nanoparticles supported on graphene fabricated by atomic layer deposition and their use as efficient synergistic microwave absorbers. Nano Research.

[3] Bergheul S, Otmane F, Azzaz M (2012). Structural and microwave absorption properties of nanostructured Fe-Co alloys. Adv. Powder Technol..

[4] Guo J (2012). One-step seeding growth of controllable Ag@Ni core-shell nanoparticles on skin collagen fiber with introduction of plant tannin and their application in high-performance microwave absorption. J. Mater. Chem..

[5] Li Z, Deng Y, Shen B, Hu W (2009). Size influence on microwave properties of Ni–Co–P hollow spheres. J. Phys. D: Appl. Phys.

[6] Zhao B, Zhao W, Shao G, Fan B, Zhang R (2015). Morphology-control synthesis of a core–shell structured NiCu alloy with tunable electromagnetic-wave absorption capabilities. ACS Appl. Mater. Inter..

[7] Wang X, Dong L, Zhang B, Yu M, Liu J (2016). Controlled growth of Cu–Ni nanowires and nanospheres for enhanced microwave absorption properties. Nanotechnology.

[8] Wen F, Zhang F, Liu Z (2011). Investigation on microwave absorption properties for multiwalled carbon nanotubes/Fe/Co/Ni nanopowders as lightweight absorbers. J. Phys. Chem. C.

[9] Yang Y, Xu C, Xia Y, Wang T, Li F (2010). Synthesis and microwave absorption properties of FeCo nanoplates. J. Alloy. Compd..

[10] Liu Q (2016). CoNi@SiO_2_@TiO_2_ and CoNi@Air@TiO_2_ Microspheres with strong wideband microwave absorption. Adv. Mater..

[11] Qiu J, Qiu T (2015). Fabrication and microwave absorption properties of magnetite nanoparticle–carbon nanotube–hollow carbon fiber composites. Carbon.

[12] Zhang Y (2015). Broadband and tunable high-performance microwave absorption of an ultralight and highly compressible graphene foam. Adv. Mater..

[13] Wang G (2012). Microwave absorption properties of carbon nanocoils coated with highly controlled magnetic materials by atomic layer deposition. ACS nano.

[14] Feng J (2016). Interfacial interactions and synergistic effect of CoNi nanocrystals and nitrogen-doped graphene in a composite microwave absorber. Carbon.

[15] Basavaraja C, Kim WJ, Do Kim Y (2011). Synthesis of polyaniline-gold/graphene oxide composite and microwave absorption characteristics of the composite films. Mater. Lett..

[16] Wang T, Wang P, Wang Y, Qiao L (2016). A broadband far-field microwave absorber with a sandwich structure. Mater. Design.

[17] Lv H, Ji G, Liang X, Zhang H, Du Y (2015). A novel rod-like MnO_2_@Fe loading on graphene giving excellent electromagnetic absorption properties. J. Mater. Chem. C.

[18] Wang C (2011). The electromagnetic property of chemically reduced graphene oxide and its application as microwave absorbing material. Appl. Phys. Lett..

[19] Singh VK (2012). Microwave absorbing properties of a thermally reduced graphene oxide/nitrile butadiene rubber composite. Carbon.

[20] Wu Q (2016). Fabrication and absorption properties based on ZnO nanocomposites adjusted by length-diameter ratio of ZnO nanorods. CrystEngComm.

[21] Wang L (2015). Fabrication of hierarchical graphene@Fe_3_O_4_@SiO_2_@polyaniline quaternary composite and its improved electrochemical performance. J. Alloy. Compd..

[22] Song J, Li J, Xu J, Zeng H (2014). Superstable transparent conductive Cu@Cu_4_Ni nanowire elastomer composites against oxidation, bending, stretching, and twisting for flexible and stretchable optoelectronics. Nano Lett..

[23] Chen S (2011). Oxidation resistance of graphene-coated Cu and Cu/Ni alloy. ACS nano.

[24] Lin J-H, Guliants VV (2012). Hydrogen production through water–gas shift reaction over supported Cu, Ni, and Cu-Ni nanoparticle catalysts prepared from metal colloids. ChemCatChem.

[25] Yusoff AN (2002). Electromagnetic and absorption properties of some microwave absorbers. J. Appl. Phys..

[26] Wu H (2015). Co^2+^/Co^3+^ ratio dependence of electromagnetic wave absorption in hierarchical NiCo_2_O_4_-CoNiO_2_ hybrids. J. Mater. Chem. C.

[27] Wu G (2015). Facile synthesis of urchin-like ZnO hollow spheres with enhanced electromagnetic wave absorption properties. Materials Lett..

[28] Che RC, Peng LM, Duan XF, Chen Q, Liang XL (2004). Microwave absorption enhancement and complex permittivity and permeability of Fe encapsulated within carbon nanotubes. Adv. Mater..

[29] Zhao J (2016). Lanthanum and neodymium doped barium ferrite-TiO_2_/MCNTs/poly (3-methyl thiophene) composites with nest structures: preparation, characterization and electromagnetic microwave absorption properties. Sci. Rep..

[30] Yang Z, Li Z, Yu L, Yang Y, Xu Z (2014). Achieving high performance electromagnetic wave attenuation: a rational design of silica coated mesoporous iron microcubes. J. Mater. Chem. C.

[31] Chen Y-H (2015). 3D Fe_3_O_4_ nanocrystals decorating carbon nanotubes to tune electromagnetic properties and enhance microwave absorption capacity. J. Mater. Chem. A.

[32] Zhao B (2015). Synthesis of flower-like CuS hollow microspheres based on nanoflakes self-assembly and their microwave absorption properties. J. Mater. Chem. A.

[33] Sun X (2013). Laminated magnetic graphene with enhanced electromagnetic wave absorption properties. J. Mater. Chem. C.

[34] Qiao L (2009). Microwave absorption properties of the hierarchically branched Ni nanowire composites. J. Appl. Phys..

[35] Zhao B, Shao G, Fan B, Zhao W, Zhang R (2015). Investigation of the electromagnetic absorption properties of Ni@TiO_2_ and Ni@SiO_2_ composite microspheres with core-shell structure. Phys. Chem. Chem. Phys..

[36] Song W-L (2016). Strong and thermostable polymeric graphene/silica textile for lightweight practical microwave absorption composites. Carbon.

[37] Qiang R (2016). Rational design of yolk-shell C@C microspheres for the effective enhancement in microwave absorption. Carbon.

[38] Meng F (2016). Design of porous C@Fe_3_O_4_ hybrid nanotubes with excellent microwave absorption. Phys. Chem. Chem. Phys..

[39] Lv H (2015). CoxFey@C composites with tunable atomic ratios for excellent electromagnetic absorption properties. Sci. Rep..

[40] Wang X, Yu M, Zhang W, Zhang B, Dong L (2014). Synthesis and microwave absorption properties of graphene/nickel composite materials. Appl. Phys. A-Mater..

[41] Qu B, Zhu C, Li C, Zhang X, Chen Y (2016). Coupling hollow Fe_3_O_4_–Fe nanoparticles with graphene sheets for high-performance electromagnetic wave absorbing material. ACS Appl. Mater. Inter..

[42] Lv H, Zhang H, Ji G, Xu ZJ (2016). Interface strategy to achieve tunable high frequency attenuation. ACS Appl. Mater. Inter..

[43] Pawar SP, Gandi M, Saraf C, Bose S (2016). Exceptional microwave absorption in soft polymeric nanocomposites facilitated by engineered nanostructures. J J. Mater. Chem. C.

[44] Jiang W, Wang Y, Xie A, Wu F (2016). Microwave absorption of a TiO_2_@PPy hybrid and its nonlinear dielectric resonant attenuation mechanism. J. Phys. D: Appl. Phys..

[45] Chen R (2013). Co-percolating graphene-wrapped silver nanowire network for high performance, highly stable, transparent conducting electrodes. Adv. Funct. Mater..

[46] Wang L (2014). Synthesis and microwave absorption enhancement of graphene@Fe_3_O_4_@SiO_2_@NiO nanosheet hierarchical structures. Nanoscale.

[47] Hummers WS, Offeman RE (1958). Preparation of Graphitic Oxide. J. Am. Chem. Soc.

[48] Zhang S, Zeng HC (2010). Solution-based epitaxial growth of magnetically responsive Cu@Ni nanowires. Chem. Mater..

