# Supplementary material for: Super-light Cu@Ni nanowires/graphene oxide composites for significantly enhanced microwave absorption performance
Source: Sci Rep. 2017 May 8;7:1584. doi: 10.1038/s41598-017-01529-2 (PMC5431521; doi:10.1038/s41598-017-01529-2)
Supplement: Supplementary file 1 — Super-light Cu@Ni nanowires/graphene oxide composites for significantly enhanced microwave absorption performance [file 41598_2017_1529_MOESM1_ESM.doc]

Supplementary Information of

Super-light Cu@Ni nanowires/graphene oxide composites for significantly enhanced microwave absorption performance

Xiaoxia Wang^1^, Baoqin Zhang^2^, Wei Zhang^2^, Mingxun Yu^2^, Liang Cui^1^, Xueying Cao^1^, Jingquan Liu^1^

*1.College of Materials Science and Engineering; Institute for Graphene Applied Technology Innovation; Laboratory of Fiber Materials and Modern Textiles, the Growing Base for State Key Laboratory; Collaborative Innovation Center for Marine Biomass Fibers Materials and Textiles of Shandong Province, Qingdao University, Qingdao 266071, China.*

*2. Shandong Institute of Nonmetal Materials, Jinan 250031, China.*

*Correspondence and requests for materials should be addressed to* [*jliu@qdu.edu.cn*](mailto:jliu@qdu.edu.cn)

**
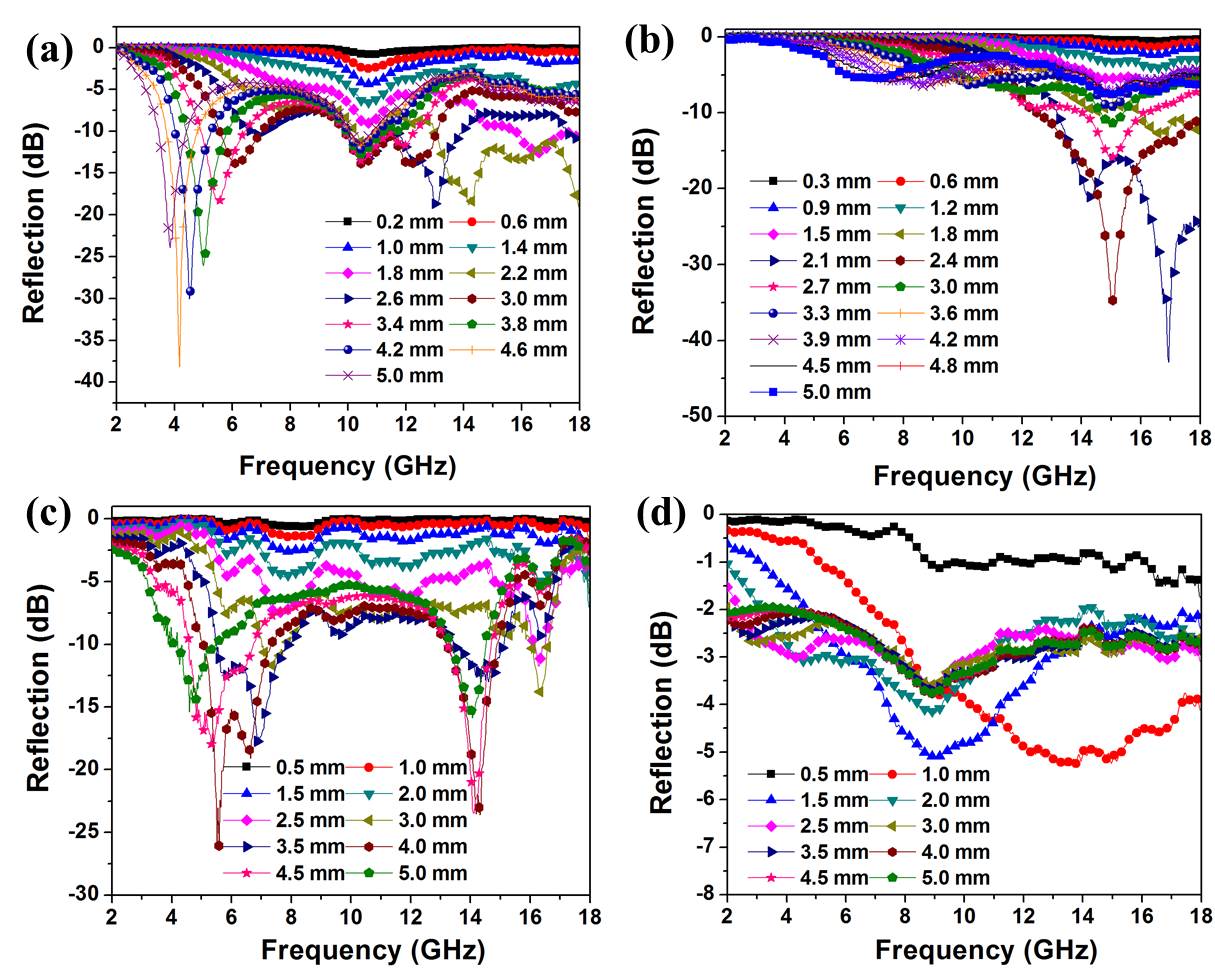
**

**Figure [S1]** Reflection loss curves of Cu@Ni NWs/GO (1:0.5) (a), Cu@Ni NWs/GO (1:1) (b), Cu@Ni NWs/GO (1:2) (c), and Cu@Ni NWs/RGO (1:1) (d) with thickness from 0.5 to 5 mm in the frequency range of 2.0-18.0 GHz.


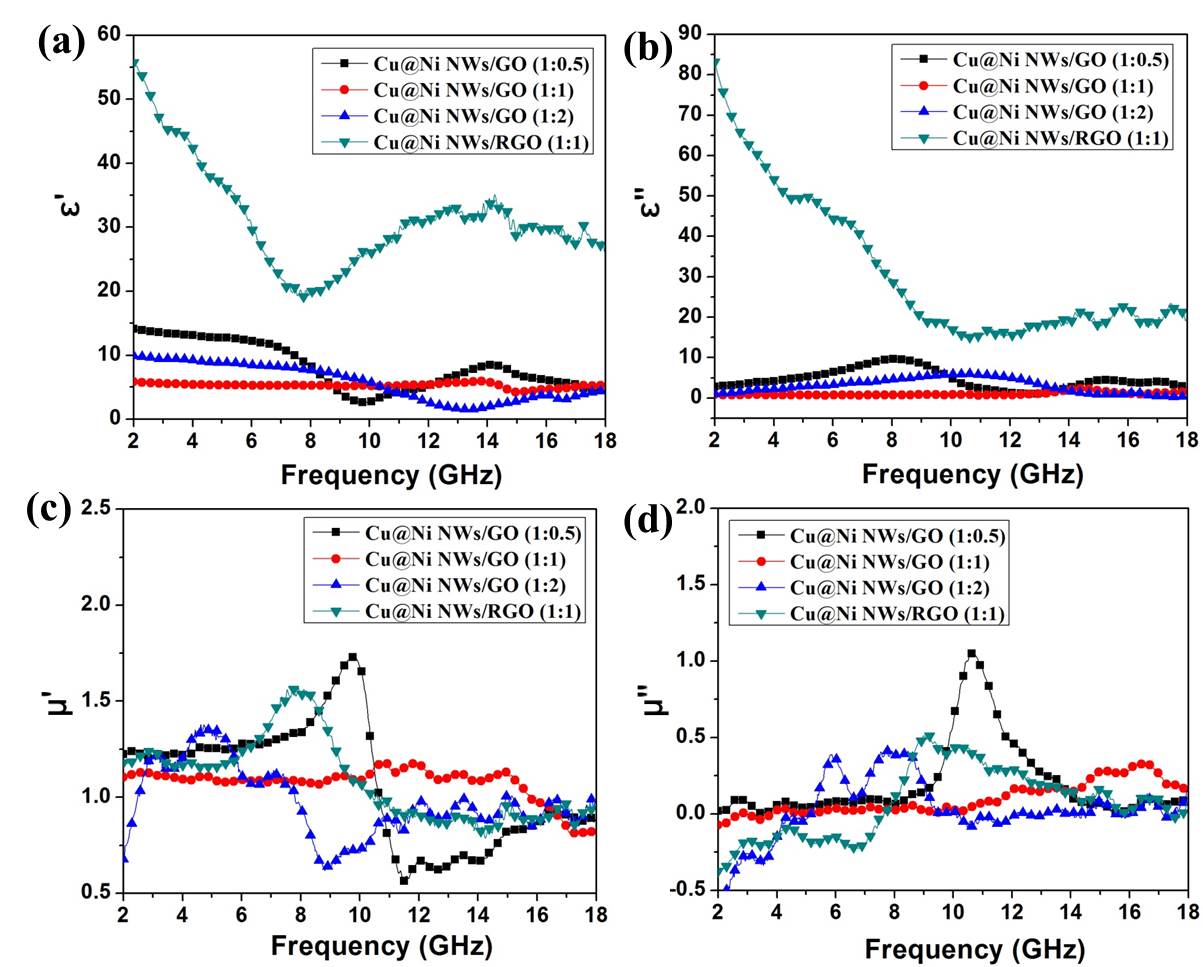


**Figure [S2]** (a-b) Real (ε') and imaginary (ε'') parts of complex permittivity and (c-d) real (μ') and imaginary (μ'') parts of permeability for Cu@Ni NWs/GO (1:0.5), Cu@Ni NWs/GO (1:1), Cu@Ni NWs/GO (1:2), and Cu@Ni NWs/RGO (1:1) in the frequency range of 2.0-18.0 GHz.


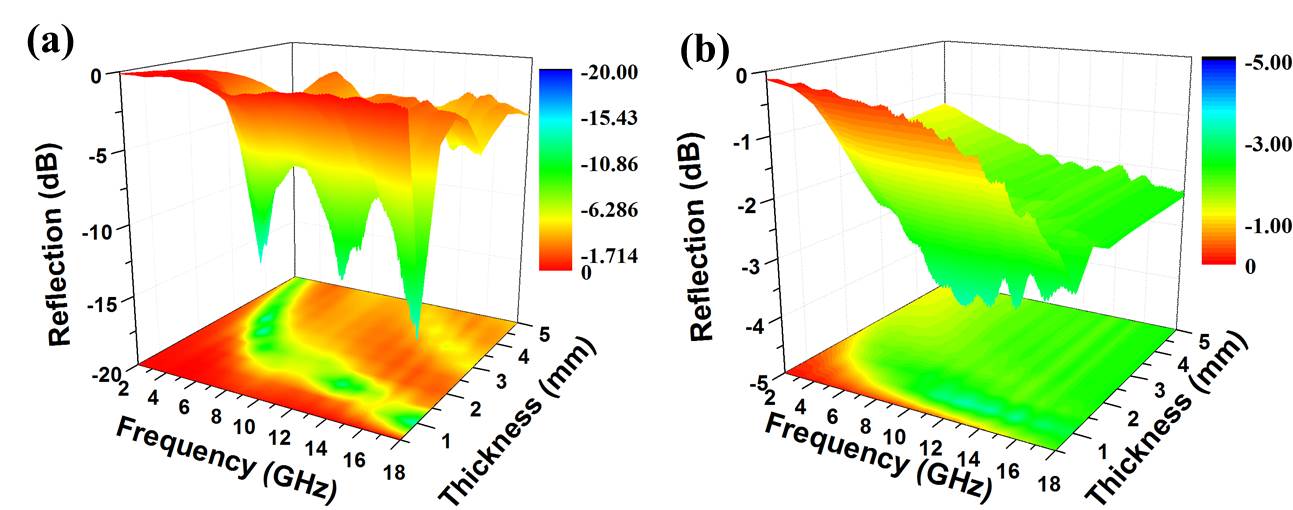


**Figure [S3]** Three-dimensional representations of RL curves for (a) Cu@Ni NWs/RGO (1:0.5) and (b) Cu@Ni NWs/RGO (1:2).


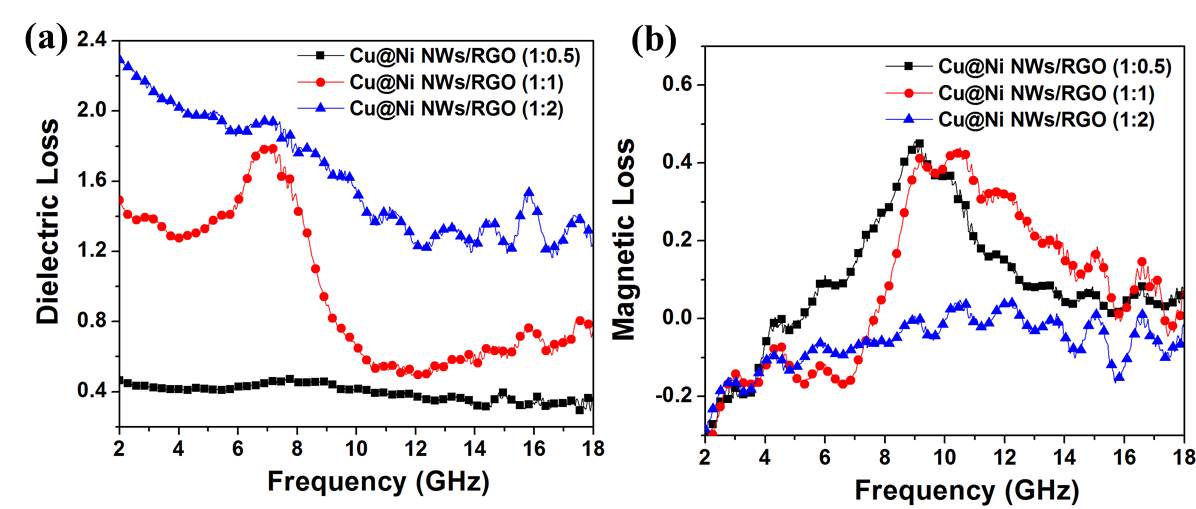


**Figure [S4]** Dielectric loss (a) and magnetic loss (b) of Cu@Ni NWs/RGO (1:0.5), Cu@Ni NWs/RGO (1:1), and Cu@Ni NWs/RGO (1:2).


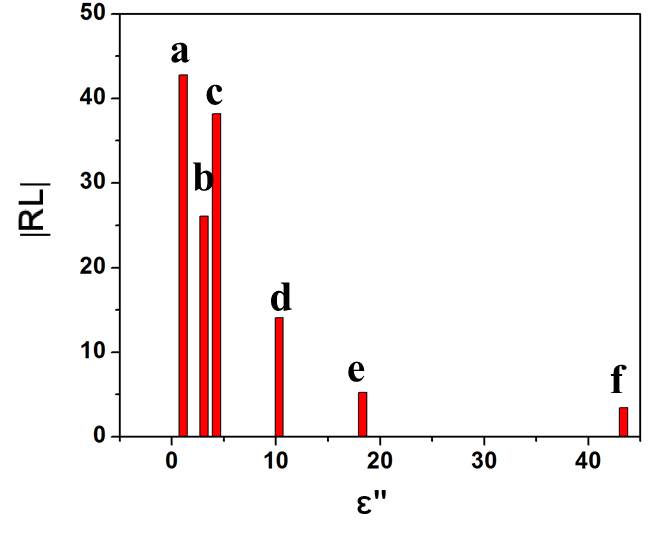


**Figure [S5]** The relationship between the values of maximum |RL| and the corresponding ε'' values for Cu@Ni NWs/GO (1:1) (a), Cu@Ni NWs/GO (1:2) (b), Cu@Ni NWs/GO (1:0.5) (c), Cu@Ni NWs/RGO (1:0.5) (d), Cu@Ni NWs/RGO (1:1) (e) and Cu@Ni NWs/RGO (1:2) (f), respectively.
